# Serum testosterone levels in male hypogonadism: Why and when to check—A review

**DOI:** 10.1111/ijcp.12995

**Published:** 2017-10-05

**Authors:** Mark Livingston, Anura Kalansooriya, Andrew J. Hartland, Sudarshan Ramachandran, Adrian Heald

**Affiliations:** ^1^ Department of Blood Sciences Walsall Manor Hospital Walsall UK; ^2^ Department of Clinical Biochemistry Heart of England NHS Foundation Trust Sutton Coldfield UK; ^3^ Department of Endocrinology and Diabetes Salford Royal Hospital Salford UK; ^4^ The School of Medicine and Manchester Academic Health Sciences Centre University of Manchester Manchester UK

## Abstract

**Aim:**

Although “late onset hypogonadism”, a condition that includes low testosterone and symptoms, is common in men over the age of 40 years, diagnosis is not clear cut amongst non‐specialists. It is the aim of this review to provide an up to date picture of how this state should be diagnosed and managed.

**Methods:**

We aim to describe how primary and secondary hypogonadism should be excluded before the diagnosis of late onset hypogonadism is reached. As laboratory testosterone measurements are essential the current pitfalls such as inappropriate sample collection and the use of population derived reference ranges are expanded. We review current evidence to determine associations between late onset hypogonadism and morbidity/mortality and benefits following testosterone replacement therapy.

**Results:**

A review of the current evidence shows that late onset hypogonadism is associated with a worse metabolic state and increased mortality. Longitudinal studies have suggested that significant reductions in both symptoms and mortality are seen, especially in patients with type 2 diabetes.

**Discussion:**

This review highlights the importance of diagnosing late onset hypogonadism due to its association with morbidity/mortality and benefits following testosterone replacement. Thus, after making recommendations to ensure correct diagnosis we speculate whether the time has come to move away from population derived testosterone levels towards evidence based action limits.

**Review criteria**

The information for this review was gathered by appraisal of relevant publications following a search of the on‐line medical database PubMed. Three of the co‐authors (ML, AHH, SR) independently rated the relevance of the research papers and other related/cited articles. The remaining co‐authors provided further information and editorial guidance.

**Message for the clinic**

- The interpretation of a testosterone level found to be low on a sample taken at 09.00, requires a serum prolactin, LH and FSH measurement in order to rule out secondary hypogonadism. Also, sex hormone binding globulin (SHBG) measurement may help.
- It is important that multiple medical disciplines are conversant with the management of male ‘late onset hypogonadism (HG)’ due to its high prevalence and diverse presenting symptoms. Decisions have to be taken regarding testosterone replacement treatment based on the evidence base.
- An understanding of longitudinal studies of men with late onset hypogonadism following TRT across primary and secondary care has the potential to yield significant morbidity and mortality benefits for our patients.

## 1 CHECKING TESTOSTERONE LEVELS IN MEN: WHY IT IS IMPORTANT

The role of the clinical laboratory is to provide tests that together with clinical judgement aid in the diagnosis and management of disease. Thus, clear indications for requesting and performing laboratory investigations are essential. Tests should not be requested due to availability (ie, random screening). It is also essential that the requesting clinician has a grasp of the clinical implications of the generated laboratory results. An underlying principle of endocrinology is that hormone levels must be judged to be appropriate or not, taking into consideration the clinical picture of the individual patient as opposed to rigidly sticking to traditional population distribution derived reference ranges. Furthermore, the generation of outcomes from research studies will impact on patient management.

Considerable confusion and unsubstantiated myths appear to affect both clinical and laboratory domains, where male testosterone levels are concerned. The objective of this review was to provide clarity and structure when requesting testosterone in men and managing hypogonadism (HG). Hence, we categorise the reasons for requesting testosterone into four areas: (i) diagnosis of secondary HG: pituitary/hypothalamic disease; (ii) improving patient fertility; (iii) diagnosis of primary HG and (iv) late onset HG (also known as testosterone deficiency or “adult onset HG”). In our view, attempts to replace late onset HG with the term “functional HG” will only provide greater confusion as there can be a “functional” deficiency in both primary and secondary hypogonadism. This review will focus on the diagnosis/management of primary/secondary HG in brief and late onset HG in depth to alleviate some of the prevailing confusion regarding both diagnosis and treatment.

Testosterone, the most important androgen produced by the testes, plays an integral role in men's health.^1^ Androgens act at several sites in the sexual response system: within the CNS, peripheral nitrergic nerves and corpora cavernosa. Androgen deficiency may be associated with decreased libido, erectile dysfunction (ED) and insensitivity to phosphodiesterase type 5 (PDE5) inhibitor treatment.^2^

## 2 DIAGNOSIS OF SECONDARY HYPOGONADISM (PITUITARY/HYPOTHALAMIC DISEASE)

Anterior pituitary pathology can be due to deficiency or hypersecretory states, with clinical symptoms and signs attributed mainly due to altered levels of hormones produced by the end organs under its regulatory control. Deficiency state could be either generalised or axis specific and often caused by non‐functional pituitary adenomas, craniopharyngiomas, metastases, inflammatory diseases, haemorrhage and infarction. Haemochromatosis (interestingly also a cause of primary HG), sarcoidosis as well as rarer infiltrative disorders can also result in pituitary damage. When hypopituitarism is diagnosed without an underlying cause being detected it is classified as “idiopathic” hypopituitarism.

Luteinizing hormone (LH) secreted by the anterior pituitary in response to hypothalamic gonadotrophin releasing hormone (GnRH), stimulates the interstitial (Leydig) cells to produce testosterone. Although testosterone has some direct end‐organ activity, its major effects appear mediated by active metabolites following peripheral conversion into oestradiol (brain and bone) and 5α‐dihydrotestosterone (in other tissues). Increased testosterone levels result in negative feedback suppressing GnRH and LH. It is important that any suspicion of pituitary or hypothalamic disorder must be referred urgently to an endocrinologist. Thus, it is essential to be aware of the symptoms and signs of pituitary/hypothalamic disorders, some of which are non‐specific and include hypotension, weight loss, hypoglycaemia, hypothyroidism and HG.

It is important to check LH, follicle‐stimulating hormone (FSH) and prolactin to differentiate secondary (pituitary/hypothalamic) from primary HG.^3^, ^4^, ^5^ Elevated FSH levels indicate Sertoli cell failure and low FSH is consistent with hypopituitarism. Elevated FSH levels are also associated with ageing in men. LH levels below the laboratory reference range are often consistent with secondary hypogonadism. Consequently, measurement of FSH and LH may assist in differentiating primary from secondary HG. Both gonadotrophins are usually raised in primary HG (typically >10 iu/L) while testosterone may or may not be low, depending on the progression of the gonadal failure. Hypothalamic/pituitary disease affecting the GnRH/LH/FSH axis usually presents as low testosterone (<9 nmol/L) without an appropriately increased gonadotrophin level or response on dynamic function testing. Referral to a specialist endocrinology clinic should be considered if the results are abnormal, inappropriate or difficult to interpret.

**Figure 1:**
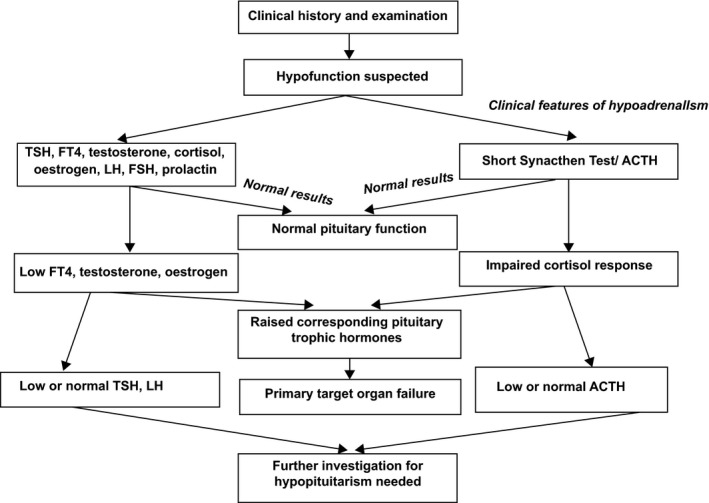
A plan of investigation when hypopituitarism is suspected. FT4, free thyroxine; TSH, thyroid‐stimulating hormone; LH, luteinizing hormone; FSH, follicle‐stimulating hormone; ACTH, adrenocorticotrophic hormone

If hypopituitarism is suspected, the entire anterior pituitary profile should be investigated (it is perhaps best to consider the hormonal deficiencies by both region of secretion and individual axes which is not within the scope of this review), including prolactin (high with some tumours, drug treatments and stress, while low in other instances), 9 am cortisol, thyroid‐stimulating hormone, free thyroxine, insulin‐like growth factor‐1, LH, FSH and repeat testosterone. A temporary hypogonadotrophinaemia may result as a response to severe stress. This can be differentiated from true hypopituitarism by the 9 am cortisol—in a stress response cortisol will be physiologically elevated. It is important to be aware that other conditions exist that demonstrate similar biochemical presentations to hypopituitarism. Diagnosing pituitary disease requires experience with knowledge of endocrine pathways as the clinical state of the patient has to be matched to the individual hormone levels followed by a cascade of dynamic function tests and imaging. As hypopituitarism is diagnosed by examining the global endocrine function and specific pituitary axes, we present a simplified investigation pathway in Figure 1. Suggested investigations for endocrine diseases are presented below.

### 2.1 The consequences of hyperprolactinaemia

Hyperprolactinaemia is associated with ED, loss of libido and anorgasmia.^6^ It is frequently accompanied by androgen deficiency since elevated prolactin levels suppress LH production leading to hypogonadism. Hyperprolactinaemia should be excluded by checking prolactin concentration in all men with diminished sexual desire. Moderate elevation of prolactin levels (<1000 mU/L) is unlikely to cause ED^7^ although it may affect libido and orgasm.^8^

Hyperprolactinaemia may result from the following:

- Medical and physical stress
- Drugs (notably neuroleptics and anti‐emetics)
- Prolactin‐secreting pituitary tumour
- Chronic renal failure

A misdiagnosis of hyperprolactinaemia can be due to the presence of macroprolactin or “big‐big” prolactin. This is a heterogenous complex of prolactin and immunoglobulin and is the cause of apparent hyperprolactaemia in about 20% of cases.^9^ Macroprolactin is detected and contributes to total prolactin measured by most commercial immunoassays, and its presence should be considered in all cases of mild to moderate hyperprolactinaemia. Macroprolactinaemia is diagnosed by re‐assaying prolactin following precipitation with polyethylene glycol and most clinical laboratories have protocols in place to check for this when prolactin levels are above the method dependent cut off (approximately 700–1000 mU/L).^9^ To avoid unnecessary investigations, baseline prolactin levels should be measured before initiating certain long‐term treatments (eg, antipsychotics). Patients with persistent and unexplained hyperprolactinaemia should be referred to an endocrinologist.

## 3 DIAGNOSIS OF PRIMARY HYPOGONADISM

This is also known as primary testicular failure and common causes include the following:

- Klinefelter's Syndrome: the extra X chromosome results in abnormal development of the testicles leading to underproduction of testosterone.
- Undescended testicles: one or both of the testes may not descend at birth, although often correcting itself within the first few years of life without intervention. If the testes remain undescended reduced secretion of testosterone can manifest.
- Mumps orchitis: in the event of infection during adolescence or adulthood, long‐term testicular damage may be evident with decreased testosterone production.
- Hemochromatosis: this can lead to both primary and secondary HG.
- Testicular trauma
- Chemotherapy or radiation therapy: this can interfere with both testosterone and sperm production and although the effects of both treatments are often short‐term, permanent infertility can manifest.

## 4 CLINICAL ASSESSMENT OF HYPOGONADISM

The presence of sexual symptoms (eg, ED, loss of libido) and generalised symptoms of HG, such as decrease in beard or body hair growth, decrease in muscle mass, gynaecomastia, osteoporosis must be ascertained on history taking. Lower testosterone levels are more likely to be associated with more clinical features of HG. Physical examination, assessment of body mass index, the waist‐hip ratio (or sagittal abdominal diameter), body hair, male pattern hair loss, presence of gynaecomastia and testicular size (measured with an orchidometer or ultrasound) and a structural examination of the penis as well as a digital rectal examination of the prostate should be included in the assessment. As described above, in the event of HG, secondary (pituitary‐dependent) and primary causes need to be excluded.

## 5 DIAGNOSIS OF LATE ONSET HYPOGONADISM

Late onset HG is defined as a combination of sexual symptoms and serum total testosterone levels <12 nmol/L.^10^ Studies have shown this age‐related male HG to affect metabolic prognostic parameters of ill health in type 2 diabetes (T2DM) and morbidity/mortality risk in adult men (eg, CVD and all‐cause mortality). Lower testosterone levels appear to correlate with more severe ED.^11^ The validated self‐administered 15 item International Index of Erectile Function (IIEF) questionnaire examines five domains: (i) erectile function, (ii) intercourse satisfaction; (iii) orgasmic function; (iv) sexual desire and (v) overall satisfaction.^12^ These domains appear affected at varying testosterone levels: loss of libido, depression in non‐obese men and decreased erectile function are more frequent at total testosterone levels <15 nmol/L, <10 nmol/L and <8 nmol/L, respectively.^13^

International guidance documents^14^, ^15^, ^16^ state that measurement of testosterone levels is mandatory in men with ED. Circulating testosterone levels are now routinely measured in men with symptoms of pituitary disease/primary HG attending endocrine, diabetes, metabolic and sexual medicine clinics, and increasingly in primary care, to investigate HG. In 2017, the American Association of Clinical Endocrinologists and the American College of Endocrinology recommended testosterone measurement in all men with type 2 diabetes, a body mass index greater than 30 kg/m2 or a waist circumference more than 102 cm. The advice of an endocrinologist or a specialist in sexual dysfunction is necessary where there is doubt about the cause and appropriate management of the HG.

### 5.1 CAG repeats and the androgen receptor

The polymorphic number of CAG repeats within the androgen receptor gene is inversely associated with the transcriptional activity of target genes.^17^ This polymorphism might thus influence testosterone effects on body fat content and serum concentrations of leptin and insulin. The direct and indirect role of androgens within the metabolic syndrome should become clearer if this genetically determined effector is taken into account.

A low number of CAG repeats had been independently associated with protective parameters such as low body fat mass and plasma insulin, as well as with adverse parameters such as reduced high‐density lipoprotein cholesterol concentrations. This suggests that a potential role of this polymorphism in modulating androgen effects on cardiovascular disease (CVD) risk factors. The pathophysiological mechanisms appear complex with an impact similar to that of androgens, dependent on exogenous cofactors.^17^ Determination of the number of CAG repeats may come to be important in planning treatment for hypogonadal men, due to possible insensitivity to testosterone replacement therapy (TRT).

### 5.2 Effect of other comorbidities on testosterone levels

Testosterone levels decline with age, but only between 6% and 12% of men have been described as developing late onset HG.^18^, ^19^, ^20^ There are a large number of comorbid conditions which have be associated with low serum testosterone,^21^ including heart failure, vascular disease, osteoporosis, dyslipidaemia, T2DM, metabolic syndrome, obesity and depression.^19^, ^22^, ^23^

Serum testosterone levels differ by ethnic group.^24^ The EMAS study^20^ found that low testosterone levels were related to obesity and the metabolic syndrome (that increase with age), not just age alone, although sex hormone binding globulin (SHBG) levels increase with age with consequent decrease in free testosterone levels.^25^ A total testosterone level <8 nmol/L has been proposed as a putative risk stratifier in the risk assesment for T2DM, CVD and all‐cause mortality.^26^ Patients with late onset HG should be considered for TRT and their symptoms monitored to assess benefit. There is a probable association between low vitamin D levels and the metabolic syndrome, including its classifying and associated factors, CVD and mortality.^27^ However, the precise nature of this relationship in subgroups (eg, gender, age groups, ethnicity, etc.) is unclear. An association of secondary and compensated HG with vitamin D deficiency has also been reported.^28^

Thus, far from being a benign consequence of ageing, HG has important and potentially harmful metabolic consequences including significantly increased CVD risk.^2^, ^29^ In men with T2DM, higher SHBG and lower free testosterone levels have been strongly associated with increased mortality.^25^ The link between late onset HG, ED and CVD/mortality is clear,^30^, ^31^, ^32^ but its management (with TRT) remains controversial with some authors suggesting that there is no benefit,^33^, ^34^ but there is a gathering body of evidence that TRT in male late onset HG does reduce CVD event rate and mortality.^32^, ^35^, ^36^, ^37^

## 6 LABORATORY INVESTIGATIONS IN LATE ONSET HYPOGONADISM

### 6.1 Total testosterone

A high proportion of serum testosterone (60%) is bound to SHBG with 38% loosely bound to albumin and other binding proteins, whilst the remaining proportion is “free” or unbound, ie, considered the physiologically active form in the body. As testosterone binds strongly to SHBG, it is the free and albumin‐bound testosterone that is available for biological action.^1^, ^38^ Thus, free testosterone is dependent on the total amount of SHBG present. Although serum free testosterone is perhaps a more reliable measure of androgen status, only serum total testosterone is available routinely. Circulating free testosterone can be estimated using online calculators (see section on free testosterone below). We recommend that both measurement of total serum testosterone and provision of calculated free testosterone should be available to the clinician, particularly when there is uncertainty in the diagnosis/management.

Circadian variation in testosterone release is the seen with peak adult testosterone levels occurring in the early morning around 09:00 am, while the lowest values (up to 60% lower) are found in the evening.^38^ Thus, samples for testosterone assay should be drawn between 08:00 and 11:00 am, since there is a degree of diurnal variation in the circulating testosterone level.

There is significant variation in laboratory reference ranges quoted for total testosterone across the UK, with some laboratories applying age‐related reference ranges. Ranges are often historical or based on those derived by the assay manufacturers (that need to be reviewed with any change in laboratory analytical equipment). Male testosterone levels decline with age^39^ gradually decreasing with each decade after the age of 40 years, leading to late onset HG. Taking the mean testosterone at age 40 years as 100%, the expected relative levels are given in Table 1. In older males, these levels can be used as a guide to when the testosterone level is borderline low. There are, however, a variety of causes of a low testosterone apart from age. If age‐related ranges are not quoted, this could increase the numbers of apparently abnormal low results in older men, with an impact on further investigation and treatment.

**Table 1:** Mean testosterone levels for each decade of life after the age of 40 y^a^

Age (y)	Percentage (%)	Lower limit normal (adjusted) nmol/L
40	100	9.4
50	93	8.7
60	85	8.0
70	79	7.4
80	72	6.8
90	67	6.3

a Taking the mean testosterone at age 40 y as 100%, the expected relative levels are given.

However, there is significant evidence that reference ranges based on population distributions should not be used, as in the case of other tests such as glycated haemoglobin A1c (HbA1c) and low‐density lipoprotein cholesterol where action limits based on evidence have replaced reference ranges. However, the lack of standardisation and current imprecision of existing testosterone assays (particularly at the lower end) makes the use of definitive cut off values for HG not possible; hence, adoption of equivocal ranges around 8–12 nmol/L together with clinical judgement.

Ideally, if the circulating total testosterone level is low on first measurement (12 nmol/L or less), the test should be repeated after 2 weeks as testosterone is released in a pulsatile manner and the value obtained from a single test may be misleading. It is important to ensure that the sample was definitely taken in the morning and in the event of timing uncertainty, a further repeat on 9 am sample would be necessary. Moreover, serum testosterone levels in men should be ideally checked in the fasting state on a morning sample, but non‐fasting levels are acceptable.^15^, ^40^

There is some difference across the literature^16^, ^41^ in relation to the cut points in terms of total and free testosterone and how they relate to the syndromal definition of HG. However, our view is that, in general, hypogonadal men with a total serum testosterone that is consistently ≤12 nmol/L with symptoms might benefit from a trial of TRT according to current guidelines.^14^, ^15^, ^42^ In a recent study of men with T2DM, where a low total testosterone was defined as <10.4 nmol/L, those on TRT for at least 2 years demonstrated reduced all‐cause mortality independent of other comorbidities and therapies.^32^ A study by Hackett et al. demonstrated a similar outcome in men with T2DM and total testosterone <12 nmol/L on TRT, this effect shown to be independent of statin and PDE5 inhibitor treatment.^10^, ^36^ The reduced mortality following TRT has also been observed in an observational cohort of non‐diabetic middle‐aged men with low total testosterone levels (≤8.7 nmol/L) and high chronic medical morbidity.^35^

As stated previously, debate exists as to the threshold testosterone in men with HG that requires TRT. However, in our view overt HG (defined as the presence of clinical symptoms of HG (eg, sexual dysfunction) and total testosterone <8 nmol/L) most definitely requires treatment. In the presence of one or more borderline results, adding an SHBG (and serum albumin) to enable estimation of the circulating free testosterone can add supportive information to the total testosterone levels. Furthermore, SHBG should also be measured in men with conditions that may affect its concentration (see section 6.3).

### 6.2 Free testosterone

Accurate measurement of free testosterone is technically difficult to achieve and basic calculations using total testosterone/SHBG (eg, free androgen index) have been discredited as assessments of free testosterone function. A more robust method to estimate free testosterone levels is based on the Vermeulen equation which includes total testosterone, SHBG and albumin levels in the algorithm.^42^, ^43^ Example calculators are available at: www.pctag.uk/testosterone-calculator (default UK units; last accessed: 17/06/2017) and http://www.issam.ch/freetesto.htm (last accessed: 17/06/2017) with a free mobile phone App version also available (“T Calc”).

In practice, in older men with suspected HG (presenting with ED) with a low or borderline‐low total testosterone level, SHBG measurement may be considered at the initial visit to reduce number of visits and prevent delays to treatment. In men with a total testosterone >12 nmol/L and symptoms, an estimated circulating free testosterone <0.225 nmol/L (<225 pmol/L) suggests the patient might benefit from a trial of TRT for hypogonadism.^14^, ^15^ Here, the estimated free testosterone potentially helps the decision to give TRT.

### 6.3 Sex hormone binding globulin

Factors that are known to decrease SHBG concentration include androgens (including anabolic steroid use), obesity, hyperinsulinism, metabolic syndrome, T2DM, hypothyroidism, glucocorticoids and atorvastatin treatment. SHBG levels are known to increase with ageing, smoking, liver cirrhosis, HIV, thyrotoxicosis and rosiglitazone treatment.^44^

## 7 INITIAL INVESTIGATIONS IN MEN PRESENTING WITH HYPOGONADISM

Initial investigations should assess common contributing factors, as well as low serum testosterone levels, which may give rise to male HG symptoms and reduce fertility/libido.^3^, ^4^, ^5^ A total testosterone above the reference range suggests possible androgen excess. Anabolic steroids should be considered as a cause of ED, gynaecomastia and testicular atrophy. Following anabolic steroid use, serum gonadotrophins are expected to be suppressed. Non‐testosterone anabolic steroids usually do not cross‐react in the testosterone assay, giving a biochemical picture similar to secondary HG. In the event of testosterone being the steroid of abuse, elevated testosterone and suppressed gonadotrophins are expected. With this pattern repeat testosterone with a request for LH, FSH, SHBG and thyroid function tests would be appropriate.

A testosterone level between 13–28 nmol/L in most cases suggests adequate gonadal function with no further testing required. Guidance states that offering testosterone replacement therapy TRT to men with total testosterone >12 nmol/L is not indicated.

Inadequate function is possible if the total testosterone levels are between 8‐12 nmol/L. Unless already measured, SHBG (to calculate the estimated free testosterone) and the anterior pituitary hormones should be measured on all samples where the testosterone is ≤12 nmol/L. Many patients with testosterone concentrations between 8 and 12 nmol/L will not have HG and do not need to be referred to an Endocrinologist. Inadequate function becomes increasing likely the lower the result is <8 nmol/L (early morning sample) and warrants consideration of a referral to a Consultant Endocrinologist for further assessment, especially at levels <3 nmol/L.

## 8 FURTHER ASSESSMENT OR REFERRAL ACCORDING TO CIRCULATING TESTOSTERONE LEVEL

### 8.1 Total testosterone level <8 nmol/L, and/or estimated free testosterone <0.225 nmol/L (<225 pmol/L), and unequivocally abnormal LH/FSH

It is recommended^19^, ^45^ that all patients with results which strongly suggest secondary or primary HG should be referred to a specialist to elucidate the cause, assess the potential associated pituitary or testicular pathology and establish the appropriate replacement therapy. Long‐term follow‐up and monitoring of treatment may be carried out in primary care depending on the underlying pathology and treatment.

In borderline cases where the pattern is unclear, it is recommended that the tests are repeated. Patients with total testosterone <8 nmol/L and/or estimated free testosterone <0.225 nmol/L (<225 pmol/L) on two consecutive occasions require investigation of the cause of their HG.

### 8.2 Total testosterone 8‐12 nmol/L, with variable estimated free testosterone, LH and FSH results:

Many of these men will be classified as having late onset HG. Most patients with a total testosterone between 8 and 12 nmol/L will not be hypogonadal if asymptomatic, and will not require further investigation and treatment. Patients experiencing symptoms of HG require further assessment, with TRT considered if not contraindicated. It is important to confirm symptomatic benefit with adequate testosterone replacement in these individuals. If no benefit is demonstrated despite adequate testosterone levels being attained, insensitivity states (estimation of CAG repeats) must be considered. In the case of sexual symptoms, it is essential that TRT is not discontinued prematurely.

### 8.3 Total testosterone levels >12 nmol/L

Patients with two consecutive total testosterone >12 nmol/L and/or estimated free testosterone >0.225 nmol/L (>225 pmol/L) do not warrant treatment. Re‐testing after 6‐12 months may be considered in some cases.

### 8.4 Further laboratory testing

When assessing patients with potential late onset HG, it may be prudent to undertake some extra testing when assessing these patients. This could include glucose/HbA1c, liver function tests, urea and electrolytes, Full Blood Count (FBC) and serum prostate specific antigen (PSA) prior to considering TRT. Patient counseling is recommended to explain the implications of measuring serum PSA, including a discussion on the benefits and drawbacks of the test.^46^

Two prostate pathologies are associated with testosterone levels: benign prostatic hyperplasia and prostate cancer. These pathologies tend to occur after 40 years of age just when testosterone levels are seen to decrease. The evidence is that there appears to be no increased risk of prostate cancer following TRT.^16^ Prostate cancer is a dihydrotestosterone responsive tumour and much of the therapy targets testosterone/dihydrotestosterone production. Examples of anti‐androgen therapy include: (i) goserelin, a GnRH analogue to suppress gonadotrophins, producing a hypopituitary hypogonadal picture, (ii) cyproterone acetate, an androgen receptor antagonist having little effect on gonadotrophin/testosterone levels, (iii) Finasteride, a 5α‐reductase inhibitor blocking the activation of testosterone to 5α‐dihydrotestosterone.

## 9 TREATMENT OF LATE ONSET HYPOGONADISM

Despite mounting evidence that TRT is associated with benefit in late onset HG, practice has been slow to take this into account. The cause of HG should always be sought before TRT is initiated (PDE5 inhibitors can be used if ED is seen, although it is well recognised that some patients on these agents may only benefit optimally when testosterone levels are normalised). Interestingly, in a meta‐analysis by Corona et al.,^47^ nearly all studies included comprised of populations of men without “classic” hypogonadism; the resulting positive results reported in those studies indicated that symptomatic testosterone‐deficient men benefited from TRT regardless of the underlying aetiology. These results provide evidence that directly contradict recommendations to limit the use of TRT only to men with classic hypogonadism.^47^

Prior to TRT, all patients must have baseline haematocrit and PSA measured. Following TRT, a small proportion of men can demonstrate aggression and mood change, hence, it is our practice to initially offer testosterone gel in view of a more rapid response related to shorter half‐life followed by a discussion with the patient regarding conversion to a longer acting injectable preparation.

TRT is usually administered by topical gel, injection, patches or implants (but patches are no longer available in Europe). Oral testosterone replacement is not recommended due to a high first pass hepatic metabolism of testosterone. A range of well‐tolerated testosterone formulations are available, including:

- Transdermal gel (Testogel^®^, Testim^®^, Tostran^®^)
- Long‐acting testosterone undecanoate injection (every 10–12 weeks) (Nebido^®^)
- Traditional depot injection (3‐weekly) (Sustanon^®^, etc.)
- Implanted pellets (the oldest form of testosterone replacement, first used in 1938 and now rarely)

Hypogonadal men restored to the eugonadal state with testosterone replacement may experience improvements in:

- sexual and erectile function
- general wellbeing
- metabolic markers (clearly seen in men with T2DM)
- responsiveness to PDE5 inhibitors^8^
- all‐cause mortality risk

Regular monitoring is essential in men receiving TRT; this must include potential androgen‐dependent symptoms and the benefits outlined above.^48^ Aim to normalise testosterone to ≥15 nmol/L^49^ if treated with testosterone gel. Patients treated with long‐acting testosterone undecanoate intramuscular injections should have trough testosterone levels in the lower normal range (>10‐12 nmol/L) depending on symptoms. In secondary HG patients, adequacy of replacement is based on testosterone levels alone whilst in primary HG, suppressed gonadotrophins are expected in over‐treated patients and raised gonadotrophins in under‐treated patients.

Following TRT, improvement in sexual desire is relatively rapid appearing after 3 weeks of treatment, and reaching a plateau at 6 weeks^36^, ^50^. However, changes in erectile function and ejaculation may require up to 6 months of TRT to show significant improvement.^51^ A recent study^50^ by Hackett et al. (2016) found that benefit in sexual symptoms after treatment with testosterone undecanoate was evident principally in men with T2DM and HG with total testosterone levels ≤8 nmol/L and free testosterone levels ≤0.18 nmol/L, their results suggested that 30 weeks of treatment was necessary before seeing a significant improvement in erectile function. Thus, it is advocated that therapeutic trials of TRT, especially with testosterone undecanoate, should be of >30 weeks' duration, not 3 months as suggested by some guidelines.^36^, ^50^

The response to treatment should be assessed at 3, 6, 9 and 12 months after the onset of treatment, and thereafter annually, including assessment of serum testosterone, haematocrit levels (as part of a FBC profile), serum PSA and other metabolic parameters (eg, lipid profile).^16^

Bone mineral density (BMD) should be monitored only in men where it was abnormal before initiation of TRT. An increase in lumbar spine BMD may already be detectable after 6 months of TRT and may continue for 3 more years.^52^ Testosterone stimulates erythropoiesis via several mechanisms involving increased erythropoietin secretion and promoting differentiation of erythroid progenitor cells of the bone marrow. Increases in haematocrit to the supra‐physiological ranges is the most frequent side effect of TRT and may be theoretically associated with hyper‐viscosity and thrombosis and be a risk factor for cerebral ischaemia. Consequently, the effect of erythropoiesis may become evident at 3 months and peaks at 12 months.^52^ When uncertain of the implications, we recommend a discussion with a haematologist as opposed to a panic driven discontinuation of treatment. It is our local practice, we have received reassurance from our colleagues in haematology regarding haematocrit levels up to 0.56.

Following TRT, there is a marginal increase in PSA and prostate volume, plateauing at 12 months.^52^ A raised or significantly increasing PSA should warrant discussion with a urologist as opposed to a knee jerk reaction to discontinue the treatment. Androgens are essential for the development and normal function of the prostate. The physiological stimulation of prostate growth induced by TRT might in theory adversely affect the prostate. Patients with benign prostatic hyperplasia (BPH) treated with androgens had been thought to be at an increased risk for worsening of signs and symptoms, but there is no evidence at present that TRT either increases the risk of BPH or contributes to worsening of lower urinary tract symptoms (LUTS), and TRT may even improve LUTS in hypogonadal men with mild BPH.^48^, ^53^ Major concerns about the risks of TRT have focused primarily on the hyperstimulation of prostate growth and the induction of carcinoma of the prostate. The evidence is that there appears to be no increased risk of prostate cancer following TRT.^16^

## 10 CONCLUSION

The focus of this review is low testosterone levels associated with HG, a common phenomenon in middle aged and older men, i.e. late onset HG. Clinical training emphasises the importance of approaching most chronic pathologies from the clinical presentation and working backwards towards the aetiology before deciding on the management. With many testosterone based myths present in healthcare circles, we felt that it was best to approach the topic from the hormone, central to much of HG.

The requesting of testosterone should be clinically driven. Testosterone should be checked in the fasted state and when low should be repeated. Free testosterone must be calculated in certain circumstances. When low testosterone levels have been confirmed, the underlying aetiology will have to be determined: primary, secondary and late onset HG. Primary and secondary HG requires referral to appropriate specialists.

It is important that multiple medical disciplines understand the management of male late onset HG due to high prevalence and diverse presenting symptoms. Decisions have to be taken regarding TRT based on evidence which has been accumulating. We have presented some of this evidence and details of TRT and post‐treatment clinical and laboratory monitoring. An understanding of longitudinal studies of men with late onset HG following TRT across primary and secondary care has the potential to yield significant morbidity and mortality benefits.

## CONFLICTS OF INTEREST

Professor Sudarshan Ramachandran, Dr Adrian Heald and Dr Mark Livingston are occasional speakers for Besins Healthcare (UK) Ltd. This company provided funding to allow this article to be available open‐access. The sponsor had no role in the content or presentation of this manuscript.

## ETHICAL APPROVAL

Not required.
